# Dynamic serum metabolomic changes during Chinese herbal hot compress therapy for postpartum uterine involution: pathway analysis and candidate biomarker identification

**DOI:** 10.3389/fmed.2026.1791959

**Published:** 2026-04-29

**Authors:** Xinxin Yin, Xiaoyan Liu

**Affiliations:** Shijiazhuang Maternity and Child Healthcare Hospital, Shijiazhuang, Hebei, China

**Keywords:** biomarkers, Chinese herbal hot compress, longitudinal metabolomics, metabolic pathways, postpartum uterine subinvolution, traditional Chinese medicine external therapy

## Abstract

**Introduction:**

Subinvolution of the postpartum uterus is a common puerperal complication. While Chinese herbal hot compress (CHHC) therapy has been widely applied to promote postpartum uterine involution, its underlying mechanisms remain unclear.

**Methods:**

This study employed a longitudinal metabolomics approach based on UPLC-Q-TOF-MS to analyze serum samples collected from 168 patients with postpartum uterine subinvolution (CHHC group, n = 86; routine care group, n = 82) at baseline (T0), day 3 (T1), and day 7 (T2) of treatment.

**Results:**

OPLS-DA analysis revealed that the metabolic profiles of the two groups were highly overlapping at T0 and gradually diverged as treatment progressed, with the most significant separation observed at T2. A total of 83 differential metabolites were identified based on the criteria of VIP > 1.0, p < 0.05, and |log₂(Fold Change)| > 0.58, among which 21 core metabolites exhibited four distinct dynamic patterns: sustained increase, sustained decrease, rise-then-fall, and fall-then-rise. KEGG pathway enrichment analysis indicated that the differential metabolites were primarily associated with arachidonic acid metabolism, glycerophospholipid metabolism, amino acid metabolism, and the tricarboxylic acid cycle. Spearman correlation analysis identified four candidate biomarkers—LysoPC(18:1), arachidonic acid, L-tryptophan, and citric acid—and the combined model achieved an AUC of 0.913. Notably, both pathway enrichment and biomarker diagnostic performance showed progressive enhancement from T1 to T2.

**Discussion:**

This study is the first to characterize the dynamic metabolic changes associated with the “warming meridians and activating blood” effects of CHHC therapy from a dynamic metabolomics perspective, providing preliminary molecular evidence for evaluating the efficacy of traditional Chinese medicine external therapies.

## 1 Introduction

Subinvolution of the uterus is a common complication during the puerperium, with an incidence of approximately 5–15%, which can lead to adverse outcomes, such as postpartum hemorrhage, intrauterine infection, and chronic pelvic pain (1). Western medical treatment relies primarily on oxytocin administration; however, some patients respond inadequately, highlighting the clinical importance of identifying safe and effective adjunctive therapies. In recent years, various physical therapies have been explored to promote postpartum uterine involution. Studies have demonstrated that low-intensity focused ultrasound effectively promotes postpartum uterine involution and improves maternal outcomes (2). Research has also indicated that auricular acupressure can enhance uterine involution following cesarean section and shorten the duration of lochia (3).

Chinese herbal hot compress (CHHC), as a representative external therapy in traditional Chinese medicine (TCM), exerts therapeutic effects through the dual action of heat and pharmacological constituents, achieving the functions of “warming meridians to dispel cold and activating blood to resolve stasis.” This therapy has been widely applied in clinical practice to promote postpartum uterine involution. A case report on penetration moxibustion for postpartum uterine subinvolution has confirmed the clinical value of TCM external therapies (4). A systematic review has indicated that TCM-assisted interventions can effectively improve various puerperal symptoms, such as postpartum lactation (5). Although existing clinical studies have confirmed the efficacy of CHHC, the molecular mechanisms underlying its therapeutic effects remain unclear, and objective efficacy evaluation indicators are lacking, which limits its clinical application and promotion.

Metabolomics enables a comprehensive capture of metabolic phenotypic changes in the body and has demonstrated unique advantages in investigating the mechanisms of action of TCM. Mass spectrometry-based metabolomics technology provides a powerful tool for elucidating the components and mechanisms of traditional Chinese medicines (6). Multi-omics-integrated analysis has been increasingly applied in TCM research, opening new avenues for clarifying the material basis and therapeutic targets of TCM (7). Research has shown that metabolomics is an effective technical approach for studying the mechanisms of TCM external therapies (8). A systematic review has indicated that metabolomics holds significant value in the diagnosis and prognostic assessment of uterine diseases (9). However, existing metabolomics studies on TCM external therapies have primarily focused on acupuncture and moxibustion, with no reports on CHHC to date. For example, Liu et al. (10) employed 1H NMR-based metabolomics to compare the therapeutic mechanisms of electro-acupuncture and moxibustion in a chronic atrophic gastritis rat model, but the study was limited to animal samples with a single post-treatment time point. Lin et al. (11) investigated herb-partitioned moxibustion in an IBS-D rat model using a similar single-time-point design. Among the few clinical studies, Li et al. (12) examined serum metabolite changes before and after acupuncture in breast cancer survivors, but adopted a single-arm design with only two time points and no parallel control group. These studies collectively highlight three research gaps: the absence of metabolomics investigations on CHHC, the predominant reliance on single-time-point designs that fail to capture temporal metabolic dynamics, and the lack of integrated approaches combining dynamic pattern classification with biomarker discovery. Longitudinal metabolomics can reveal the temporal regulatory characteristics of metabolic networks, and the integrated analysis of time-series metabolic data provides a unique perspective for biomarker discovery (13).

To address these gaps, this study employs untargeted metabolomics technology to conduct multi-time-point serum metabolomics analysis in a clinical cohort with a parallel control group, representing the first metabolomics investigation of CHHC therapy for postpartum uterine subinvolution. The contributions of this study include the following: elucidating the dynamic metabolic regulatory characteristics of CHHC across different treatment stages from a longitudinal metabolomics perspective, identifying differential metabolites associated with therapeutic efficacy as potential biomarkers, and interpreting the modern biological mechanisms underlying the “warming meridians and activating blood” effects of CHHC through metabolic pathway analysis.

## 2 Materials and methods

### 2.1 Study design and participants

This observational cohort study was conducted at the Obstetrics Department of Shijiazhuang Maternal and Child Health Hospital from January 2024 to March 2025. The inclusion criteria were as follows: (1) diagnosis of postpartum uterine subinvolution according to the diagnostic criteria in the 9th edition of “Obstetrics and Gynecology,” (2) age 20–40 years, (3) full-term singleton pregnancy, and (4) vaginal delivery or cesarean section. The exclusion criteria included the following: (1) presence of severe obstetric complications, (2) history of metabolic diseases, and (3) recent use of medications affecting metabolism. All participants provided written informed consent, and the study protocol was approved by the Hospital Ethics Committee (approval number: SJZFY-EC-2023-0216). It should be noted that this study did not include a placebo control group (e.g., a hot compress without herbal ingredients), as the distinct herbal odor of CHHC made effective blinding infeasible, and ethical concerns were raised regarding applying a known ineffective intervention to symptomatic postpartum patients. This limitation is further addressed in the Discussion.

Based on previous literature, the uterine fundal height was expected to decrease by approximately 1.5 cm in the CHHC group compared with the routine care group, with a standard deviation of approximately 2.0 cm. With a two-sided *α* of 0.05 and a power (1-*β*) of 0.80, using the two-sample independent *t*-test formula, a minimum of 70 participants per group was required. Considering an anticipated dropout rate of approximately 15%, 82 participants per group were planned for enrollment, totaling 164 participants. A total of 168 participants were actually enrolled (CHHC group, *n* = 86; routine care group, *n* = 82), meeting the statistical requirements.

### 2.2 Grouping and exposure

Participants were assigned to the CHHC group or the routine care group based on actual clinical treatment. The CHHC group received CHHC therapy in addition to routine postpartum care. The herbal formula consisted of *Artemisia argyi* (30 g), *Carthamus tinctorius* (15 g), *Angelica sinensis* (20 g), *Ligusticum chuanxiong* (15 g), and *Leonurus japonicus* (30 g), which were coarsely ground and packaged for use. The compress was applied to the lower abdomen and lumbosacral region at a temperature of 40–50 °C for 20–30 min once daily for 7 days. The routine care group received only standard postpartum care.

### 2.3 Sample collection and processing

Serum samples were collected at baseline (T0), day 3 (T1), and day 7 (T2) under fasting conditions at a fixed time (8:00–9:00 a.m.). Following collection, samples were allowed to stand at room temperature for 30 min and then centrifuged (3,000 rpm, 10 min, 4 °C). The supernatants were aliquoted and stored at −80 °C. Quality control information, such as hemolysis status and freeze–thaw cycles, was recorded.

### 2.4 Clinical efficacy evaluation indicators

The primary outcome measures included uterine fundal height and uterine volume. Uterine fundal height was measured as the distance from the upper edge of the pubic symphysis to the uterine fundus using a flexible tape by a trained midwife. Uterine volume was calculated using the prolate ellipsoid formula (*V* = *π*/6 × length × width × anteroposterior diameter) based on transabdominal ultrasonographic measurements performed by a designated sonographer blinded to group allocation, using a Mindray DC-80 ultrasound system with a 3.5 MHz convex probe.

Secondary outcome measures included lochia volume score and lochia color transition time. The lochia volume score was assessed using a 4-point scale: 0 = no lochia, 1 = scant (pad less than half saturated per 8 h), 2 = moderate (pad half to fully saturated per 8 h), and 3 = heavy (pad fully saturated within 4 h). The lochia color transition time was defined as the number of days from delivery until the lochia color changed from red (lochia rubra) to pink/brown (lochia serosa), as recorded daily by the attending nurse. All measurements were performed at T0, T1, and T2, synchronized with serum collection. Clinical efficacy at T2 was evaluated based on the degree of uterine involution and lochia resolution. The criteria were defined as follows: “markedly effective” if uterine fundal height decreased by ≥3 cm and lochia volume score decreased by ≥2 points; “effective” if uterine fundal height decreased by ≥1.5 cm and lochia volume score decreased by ≥1 point, and “ineffective” if neither criterion was met. The total effective rate was calculated as (markedly effective + effective)/total × 100%.

### 2.5 Metabolomics analysis

#### 2.5.1 Sample preparation

Serum samples were thawed at 4 °C, and 100 μL of each sample was mixed with 400 μL of pre-chilled methanol/acetonitrile (1:1, v/v) for protein precipitation. The mixture was vortexed for 30 s, incubated at 4 °C for 10 min, and then centrifuged (13,000 rpm, 15 min, 4 °C). The supernatant was dried under nitrogen and reconstituted with 100 μL of reconstitution solution, followed by additional centrifugation. The final supernatant was transferred to sample vials for analysis (14). Quality control (QC) samples were prepared by pooling equal volumes from all samples and were used to assess instrumental stability and data quality (15).

#### 2.5.2 LC–MS/MS analysis

Metabolomics analysis was performed using a Waters ACQUITY UPLC system coupled with a Thermo Q Exactive high-resolution mass spectrometer (16). Chromatographic separation was achieved using an ACQUITY UPLC HSS T3 column (2.1 × 100 mm, 1.8 μm) maintained at 35 °C with a flow rate of 0.3 mL/min. Mobile phase A consisted of 0.1% formic acid in water, and mobile phase B consisted of 0.1% formic acid in acetonitrile. The gradient elution program was as follows: 0–2 min, 5% B; 2–12 min, 5–95% B; 12–14 min, 95% B; 14–14.5 min, 95–5% B; and 14.5–17 min, 5% B for equilibration. Mass spectrometric detection was performed using an electrospray ionization (ESI) source with simultaneous acquisition in both positive and negative ion modes. The scan range was *m/z* 70–1,050 at a resolution of 70,000. The spray voltage was set at 3.5 kV (positive ion mode) and 3.0 kV (negative ion mode), and the capillary temperature was maintained at 320 °C (17).

#### 2.5.3 Data processing

Raw data were processed using Compound Discoverer software for peak identification, alignment, and extraction. Data preprocessing included missing value imputation (1/5 of the minimum value), normalization (total ion current normalization), and data filtering (removal of features with RSD > 30%).

### 2.6 Statistical analysis

Clinical data were analyzed using SPSS software. Normally distributed data were expressed as mean ± standard deviation, and between-group comparisons were performed using the *t*-test. Non-normally distributed data were analyzed using the Mann–Whitney *U* test. Repeated measures data were analyzed using repeated measures analysis of variance (ANOVA), with *p* < 0.05 considered statistically significant.

Metabolomics data were log_2_-transformed and Pareto-scaled before multivariate statistical analysis on the MetaboAnalyst 5.0 platform (18). Principal component analysis (PCA) was employed to observe overall distribution, and orthogonal partial least squares discriminant analysis (OPLS-DA) was used for between-group discrimination (19). Model validation was performed using 200 permutation tests and sevenfold cross-validation. Differential metabolites were screened based on VIP > 1.0, *p* < 0.05, and |log_2_(Fold Change)| > 0.58 (corresponding to Fold Change > 1.5 or < 0.67), with false discovery rate (FDR) correction (20). Metabolite identification was based on accurate mass and MS/MS fragmentation patterns, with searches conducted in the HMDB and KEGG databases (21). Pathway enrichment analysis was performed using the KEGG database, with *p* < 0.05 and impact > 0.1 set as the criteria for significantly enriched pathways (22). Spearman correlation analysis was employed to assess associations between metabolites and clinical indicators, and receiver operating characteristic (ROC) curves were used to evaluate the diagnostic performance of candidate biomarkers (23).

## 3 Results

### 3.1 Baseline characteristics and clinical efficacy

A total of 168 patients with postpartum uterine subinvolution were enrolled in this study, including 86 in the CHHC group and 82 in the routine care group. No statistically significant differences were observed between the two groups in baseline characteristics, such as age (28.7 ± 4.2 years vs. 29.1 ± 3.9 years), gestational age (39.2 ± 1.1 weeks vs. 39.0 ± 1.3 weeks), delivery mode (vaginal delivery/cesarean section: 52/34 vs. 48/34), and parity (primiparous/multiparous: 54/32 vs. 50/32) (*p* > 0.05), indicating comparability between the groups (Table 1).

**Table 1:** Comparison of baseline characteristics between the two groups.

Characteristic	CHHC group (*n* = 86)	Routine care group (*n* = 82)	*p*-value
Demographic characteristics			
Age (years)	28.7 ± 4.2	29.1 ± 3.9	0.515
Obstetric characteristics			
Gestational age (weeks)	39.2 ± 1.1	39.0 ± 1.3	0.279
Delivery mode (*n*)			0.682
Vaginal delivery	52	48	
Cesarean section	34	34	
Parity (*n*)			0.765
Primiparous	54	50	
Multiparous	32	32	
Baseline clinical indicators (T0)			
Uterine fundal height (cm)	15.8 ± 1.7	15.6 ± 1.8	0.453
Uterine volume (cm^3^)	178.5 ± 31.2	175.8 ± 33.6	0.586

Continuous variables are presented as mean ± SD (x̄±s), categorical variables are presented as counts (*n*). Between-group comparisons: independent samples *t*-test for continuous variables, *χ*^2^ test for categorical variables.

The clinical outcomes at each time point are summarized in Table 2. Both uterine fundal height and uterine volume decreased progressively in both groups, with significantly greater reductions observed in the CHHC group. Repeated measures ANOVA revealed significant time × group interaction effects for both uterine fundal height (*F* = 6.47, *p* < 0.01) and uterine volume (*F* = 8.91, *p* < 0.001), with *post-hoc* comparisons confirming that the between-group difference was most pronounced at T2 (*p* < 0.001). The CHHC group also showed greater improvement in lochia volume score (*p* < 0.05) and shorter lochia color transition time (4.2 ± 1.1 vs. 5.6 ± 1.3 days, *p* < 0.01).

**Table 2:** Clinical outcome measures at T0, T1, and T2.

Outcome	Time	CHHC group (*n* = 86)	Routine care group (*n* = 82)	*p*-value
Uterine fundal height (cm)	T0	15.8 ± 1.7	15.6 ± 1.8	0.453
	T1	14.2 ± 1.5	14.6 ± 1.6	0.092
	T2	12.1 ± 1.4	13.4 ± 1.5	<0.001
Uterine volume (cm^3^)	T0	178.5 ± 31.2	175.8 ± 33.6	0.586
	T1	152.3 ± 27.8	161.2 ± 29.4	0.041
	T2	118.6 ± 24.5	138.7 ± 26.1	<0.001
Lochia volume score	T0	2.4 ± 0.6	2.3 ± 0.7	0.318
	T1	1.5 ± 0.5	1.9 ± 0.6	<0.01
	T2	0.6 ± 0.4	1.1 ± 0.5	<0.001
Lochia color transition (days)	—	4.2 ± 1.1	5.6 ± 1.3	<0.01

Values are mean ± SD. Between-group comparisons at each time point: independent samples *t*-test. Repeated measures ANOVA: uterine fundal height (time × group interaction: *F* = 6.47, *p* < 0.01); uterine volume (time × group interaction: *F* = 8.91, *p* < 0.001).

Clinical efficacy at T2 was evaluated based on the degree of uterine involution and lochia resolution. The criteria were defined as follows: “markedly effective” if uterine fundal height decreased by ≥3 cm and lochia volume score decreased by ≥2 points; “effective” if uterine fundal height decreased by ≥1.5 cm and lochia volume score decreased by ≥1 point; “ineffective” if neither criterion was met. The total effective rate was calculated as (markedly effective + effective)/total × 100%.

### 3.2 Metabolomics data quality assessment and overall metabolic profiles

Quality assessment of the metabolomics data revealed that 87.3% of feature peaks had RSD < 30%, indicating good instrumental stability and reliable data quality. A total of 4,826 feature peaks were detected in positive ion mode, and 3,512 feature peaks were detected in negative ion mode.

Quality control (QC) samples were prepared by pooling equal volumes from all samples, and one QC sample was inserted after every 10 samples throughout the analytical sequence to assess instrumental stability and data quality. The QC samples clustered tightly in the PCA score plot, indicating stable and reliable instrumental performance throughout the analysis. Analysis of relative standard deviation (RSD) of feature peaks showed that 87.3% (4,213/4,826) of feature peaks in positive ion mode and 85.6% (3,006/3,512) in negative ion mode had RSD < 30%, meeting the quality requirements for metabolomics data.

**Figure 1:**
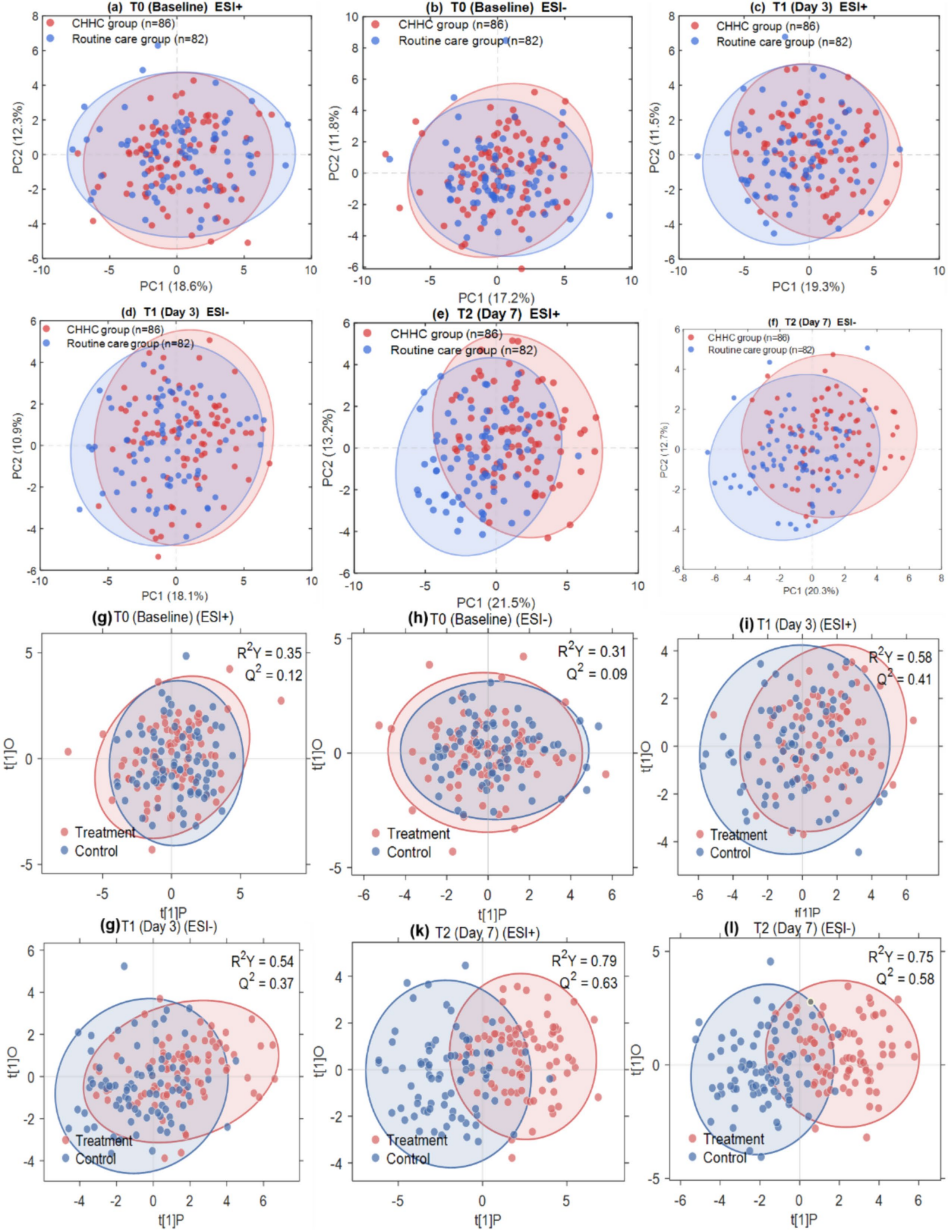
PCA **(a–f)** and OPLS-DA **(g–l)** score plots comparing metabolic profiles between the CHHC and routine care groups at T0, T1, and T2 in ESI+ and ESI− modes. Model parameters are shown in each panel. Red: CHHC group (*n* = 86); Blue: routine care group (*n* = 82). Shaded areas: 95% confidence ellipses.

PCA was first performed as an unsupervised method to visualize the overall distribution of samples without class information (Figures 1a–f). At T0, the PCA score plots showed extensive overlap between the two groups in both ESI+ (PC1: 18.6%, PC2: 12.3%) and ESI− (PC1: 17.2%, PC2: 11.8%) modes, confirming comparable baseline metabolic states. At T1, a partial separation trend emerged (ESI+: PC1: 19.3%, PC2: 11.5%; ESI−: PC1: 18.1%, PC2: 10.9%), which became more evident at T2 (ESI+: PC1: 21.5%, PC2: 13.2%; ESI−: PC1: 20.3%, PC2: 12.7%), indicating progressive metabolic divergence associated with CHHC intervention. Subsequently, OPLS-DA models were employed to analyze the differences in metabolic profiles between the CHHC group and the routine care group at each time point (Figures 1g–l). At T0 baseline, the metabolic profiles of the two groups were highly overlapping, with *R*^2^*Y* = 0.35 and *Q*^2^ = 0.12 in positive ion mode, and *R*^2^*Y* = 0.31 and *Q*^2^ = 0.09 in negative ion mode. The weak discriminatory ability of the models indicated good comparability of baseline metabolic states between the two groups. At T1 (day 3 of treatment), a separation trend began to emerge between the two groups, with *R*^2^*Y* = 0.58 and *Q*^2^ = 0.41 in positive ion mode, and *R*^2^*Y* = 0.54 and *Q*^2^ = 0.37 in negative ion mode, suggesting that the early metabolic effects of CHHC intervention had already manifested. At T2 (day 7 of treatment), the most pronounced separation was observed between the two groups, with *R*^2^*Y* = 0.79 and *Q*^2^ = 0.63 in positive ion mode, and *R*^2^*Y* = 0.75 and *Q*^2^ = 0.58 in negative ion mode, indicating significant differences in metabolic profiles between the CHHC group and the routine care group at the end of the treatment course. These findings were consistent with previous metabolomics studies on TCM external therapies (10). Model validation using 200 permutation tests showed that the *Q*^2^ intercepts for all models at T1 and T2 were negative (range: −0.18 to −0.12), well below the threshold of 0.05, indicating that the models were robust and reliable without overfitting (Supplementary Figure S1) (24). These results suggested that CHHC therapy was associated with specific changes in the serum metabolic profiles of patients with postpartum uterine subinvolution, and these changes progressively intensified with treatment duration, with between-group differences showing an increasing trend from T0 → T1 → T2.

### 3.3 Identification of differential metabolites

**Figure 2:**
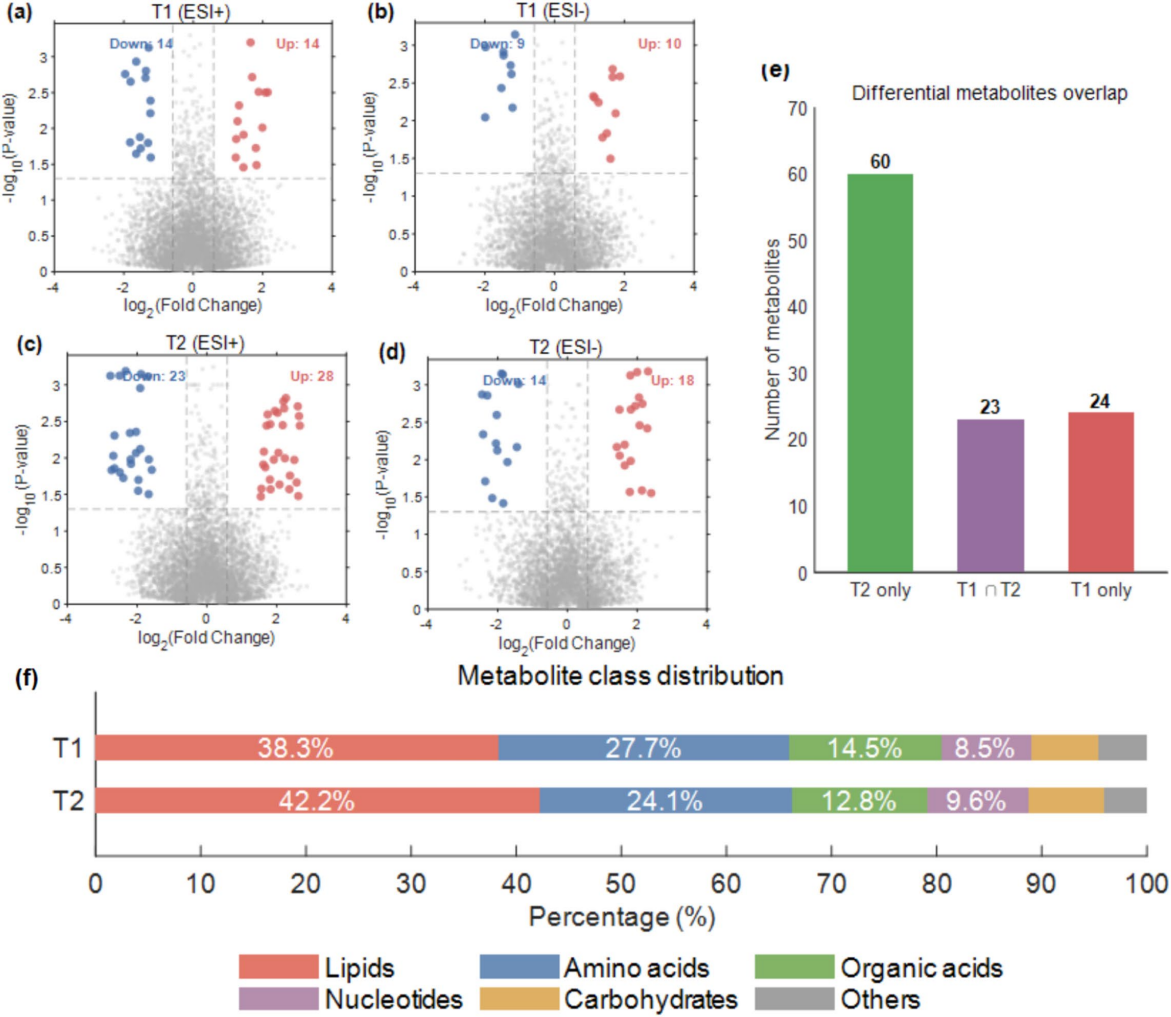
Identification of differential metabolites between treatment and control groups. **(a,b)** Volcano plots at T1, ESI+ and ESI− modes; **(c,d)** Volcano plots at T2, ESI+ and ESI− modes. Red: up-regulated; blue: down-regulated; gray: non-significant. Dashed lines indicate thresholds (|log_2_FC| > 0.58, *p* < 0.05). **(e)** Upset plot showing overlap of differential metabolites between T1 and T2. **(f)** Metabolite class distribution at T1 and T2. Differential metabolites were screened using VIP > 1.0, *p* < 0.05, and |log_2_FC| > 0.58. Key metabolites are labeled in the volcano plots.

Based on the screening criteria of VIP > 1.0, *p* < 0.05, and |log_2_(Fold Change)| > 0.58, a total of 47 differential metabolites were identified between the CHHC group and the routine care group at T1 (28 in positive ion mode, 19 in negative ion mode); the number of differential metabolites increased to 83 at T2 (51 in positive ion mode, 32 in negative ion mode), indicating that the number of differential metabolites increased with treatment duration (Figures 2a–d). Volcano plots showed that the fold change magnitudes of differential metabolites at T2 were greater than those at T1, indicating that the metabolic differences associated with CHHC intervention were more pronounced at the end of the treatment course. These findings were consistent with previous acupuncture metabolomics studies (12).

Upset plot analysis revealed that 23 differential metabolites were shared between T1 and T2, suggesting that these metabolites underwent continuous changes throughout the treatment process and may represent core target metabolites of CHHC intervention (Figure 2e). Of these, 21 showed consistent correlation directions across both stages and were retained as core metabolites for subsequent analyses (see Section 3.6). Sixty differential metabolites were unique to T2, reflecting metabolic alterations that emerged in the later phase of treatment; 24 differential metabolites were unique to T1, some of which may represent metabolites that were gradually recovered after an initial stress response. Together, these findings indicate a progressive expansion of metabolic perturbation associated with CHHC intervention from T1 to T2, with both the number and magnitude of differential metabolites increasing over time.

The class distribution of differential metabolites showed that lipids accounted for the highest proportion (T1: 38.3%; T2: 42.2%), primarily including phosphatidylcholines, lysophosphatidylcholines, and fatty acids; amino acids ranked second (T1: 27.7%; T2: 24.1%), involving phenylalanine, tryptophan, arginine, and others; organic acids accounted for 12.8–14.5%, such as citric acid, succinic acid, and other tricarboxylic acid cycle intermediates; the remainder consisted of nucleotides, carbohydrates, and other metabolites (Figure 2f). Studies have shown that alterations in lipid and amino acid metabolism are important metabolic features of TCM external therapy efficacy (25).

### 3.4 Dynamic patterns of differential metabolites

**Figure 3:**
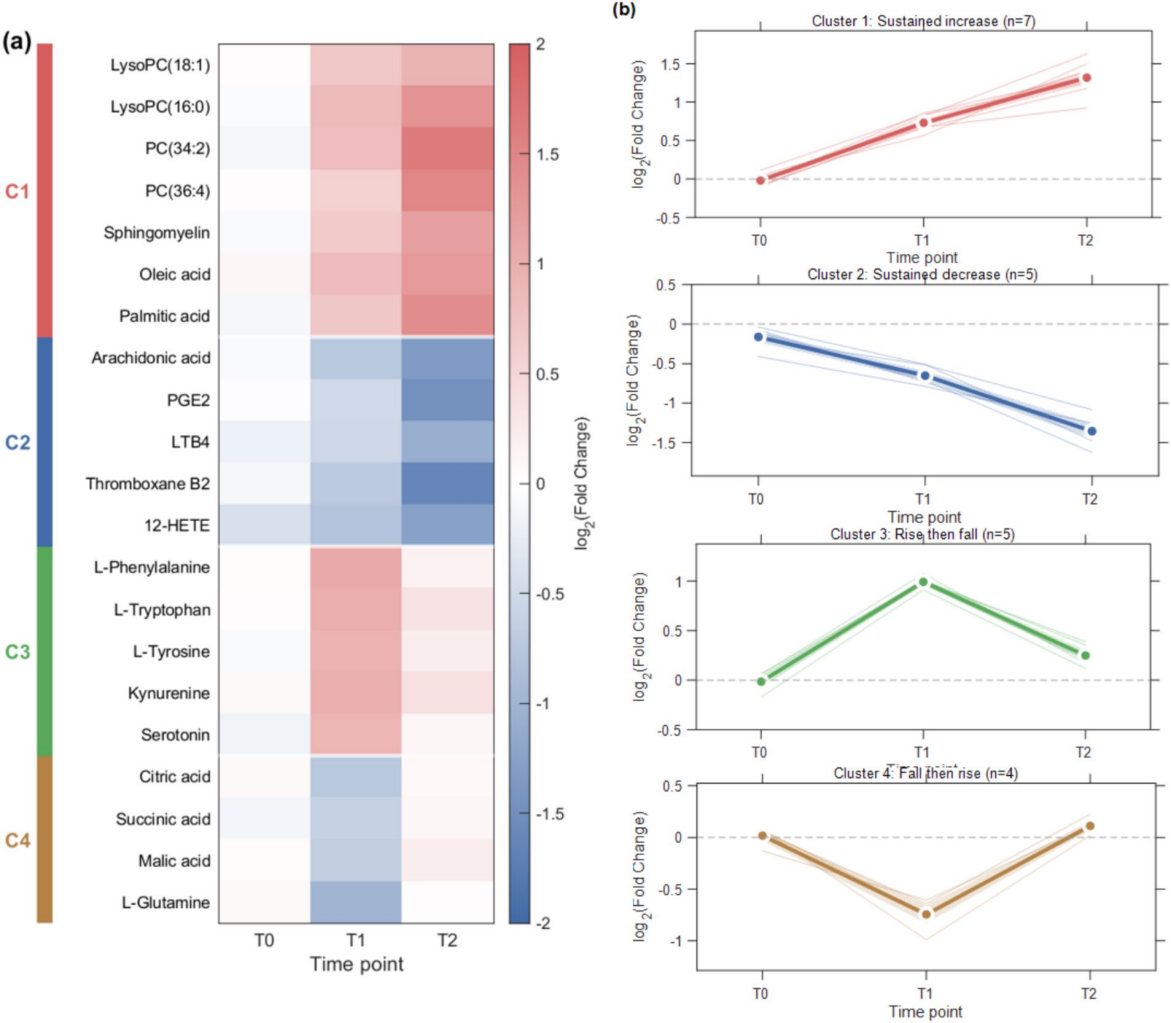
Dynamic patterns of core differential metabolites across time points. **(a)** Hierarchical clustering heatmap of 21 metabolites identified at both T1 and T2. Clustering was performed using Euclidean distance and Ward’s linkage method. The color scale represents log_2_(Fold Change) between the herbal hot compress and routine care groups. Left color bars indicate cluster assignment. **(b)** Temporal trends of each cluster from T0 to T2. Cluster 1: sustained increase (*n* = 7); Cluster 2: sustained decrease (*n* = 5); Cluster 3: rise then fall (*n* = 5); Cluster 4: fall then rise (*n* = 4).

Hierarchical clustering analysis was performed on the 21 core differential metabolites identified at both T1 and T2. Based on their expression trajectories across T0, T1, and T2, these metabolites were classified into four typical dynamic patterns (Figure 3). Cluster 1 contained seven metabolites, which exhibited a sustained increase pattern, with expression levels in the CHHC group progressively increasing relative to the routine care group from T0 → T1 → T2, primarily involving phosphatidylcholines and lysophosphatidylcholines. Cluster 2 contained five metabolites, which showed a sustained decrease pattern, with expression levels progressively decreasing from T0 → T1 → T2, mainly comprising arachidonic acid and its derivatives. Cluster 3 contained five metabolites displaying a rise-then-fall pattern, with increases from T0 → T1, followed by decreases from T1 → T2, suggesting that these metabolites may be activated in the early phase of treatment to initiate tissue repair and immune regulation processes, and then gradually declining while remaining above baseline levels. Cluster 4 contained four metabolites exhibiting a fall-then-rise pattern, with decreases from T0 → T1 followed by recovery from T1 → T2.

Longitudinal metabolomics studies have demonstrated that the identification of temporal metabolic change patterns helps reveal the dynamic regulatory mechanisms of interventions (26). Metabolites with sustained increase or sustained decrease patterns may reflect the cumulative effects of CHHC intervention, while metabolites with rise-then-fall or fall-then-rise patterns may represent adaptive regulatory processes of the body. It has been reported that clustering analysis combined with dynamic trajectory tracking can effectively identify metabolic markers associated with clinical outcomes (27). Heatmap visualization showed that the metabolic differences between the CHHC group and the routine care group were most pronounced at T2, and metabolites within each cluster exhibited highly consistent change trends, suggesting that metabolites within the same cluster may participate in similar biological processes.

### 3.5 Metabolic pathway enrichment analysis

**Figure 4:**
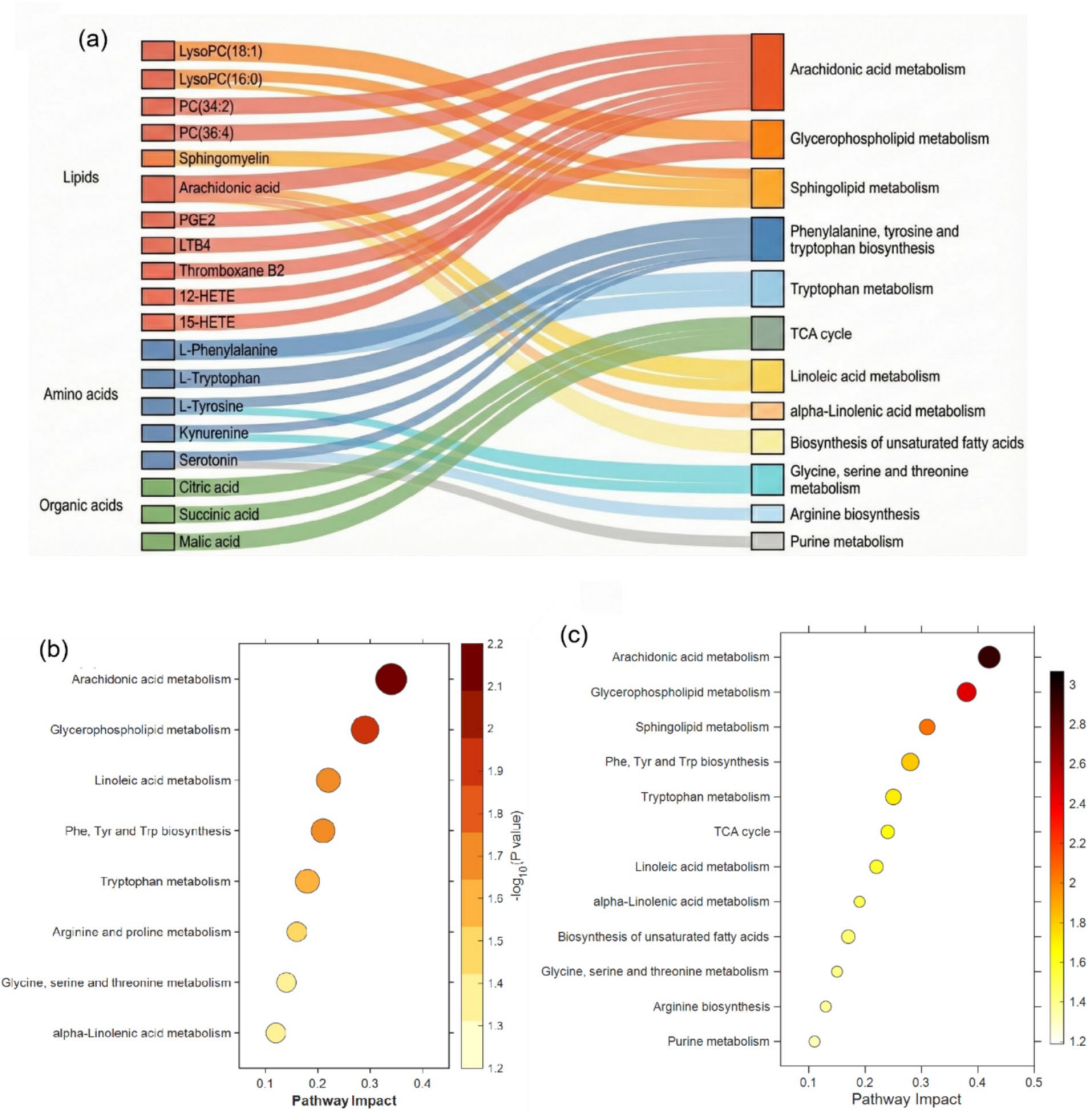
KEGG pathway enrichment analysis of differential metabolites. **(a)** Sankey diagram showing the mapping between differential metabolites (left) and KEGG-enriched pathways (right) at T2. Metabolites are grouped by chemical class (lipids, amino acids, and organic acids), and flow band colors correspond to metabolite classes. **(b)** Bubble plot of significantly enriched pathways at T1 (*p* < 0.05, Impact >0.1); 8 pathways were identified. **(c)** Bubble plot of significantly enriched pathways at T2 (*p* < 0.05, Impact> 0.1); 12 pathways were identified. In **(b,c)**, bubble size represents the number of enriched metabolites, color intensity indicates statistical significance (−log₁₀ *p-*value), and the *x*-axis represents pathway impact value.

Pathway enrichment analysis was performed using the KEGG database for differential metabolites at both T1 (47 metabolites) and T2 (83 metabolites) to reflect the dynamic changes of metabolic pathways during CHHC intervention. At T1, a total of eight significantly enriched pathways were identified (*p* < 0.05, Impact>0.1) (Figure 4b). Arachidonic acid metabolism (*p* = 0.0068, Impact = 0.34) and glycerophospholipid metabolism (*p* = 0.0125, Impact = 0.29) were already significantly enriched at T1, suggesting early activation of these pathways. Amino acid metabolism-related pathways showed relatively lower enrichment significance at T1 compared with T2, indicating that these pathways were predominantly altered in the later phase of treatment. With the screening criteria of *p* < 0.05 and pathway impact>0.1, a total of 12 significantly enriched metabolic pathways were identified (Figure 4). Arachidonic acid metabolism showed the highest enrichment (*p* = 0.0012, Impact = 0.42), involving eight differential metabolites, such as arachidonic acid, prostaglandin E2, leukotriene B4, and thromboxane B2, suggesting that CHHC intervention may exert anti-inflammatory effects by regulating the arachidonic acid cascade. Glycerophospholipid metabolism (*p* = 0.0035, Impact = 0.38) and sphingolipid metabolism (*p* = 0.0089, Impact = 0.31) involved six and four differential metabolites, respectively, mainly comprising phosphatidylcholines, lysophosphatidylcholines, and sphingomyelins, reflecting the regulatory effects of CHHC on cellular membrane lipid homeostasis.

Amino acid metabolism-related pathways were also significantly enriched. The phenylalanine, tyrosine, and tryptophan biosynthesis pathway (*p* = 0.0156, Impact = 0.28) involved five differential metabolites. Research has shown that the tryptophan–kynurenine metabolic axis plays an important regulatory role in gynecological diseases, and its metabolic disturbance is closely associated with dysmenorrhea and endometriosis (28). The tricarboxylic acid cycle pathway (*p* = 0.0234, Impact = 0.24) involved organic acid metabolites, such as citric acid, succinic acid, and malic acid, suggesting that CHHC may affect energy metabolism processes. It has been reported that patients with dysmenorrhea during the luteal regression phase exhibit significant alterations in energy metabolism pathways (29).

Comparison of pathway enrichment results between T1 and T2 revealed a progressive expansion of metabolic changes associated with CHHC intervention (Supplementary Table S1). At T1, 8 pathways were significantly enriched, which increased to 12 at T2. Notably, arachidonic acid metabolism and glycerophospholipid metabolism were consistently enriched at both time points with increasing significance (T1 → T2: arachidonic acid metabolism *p* = 0.0068 → 0.0012, Impact = 0.34 → 0.42), while pathways such as the tricarboxylic acid cycle and sphingolipid metabolism became significantly enriched only at T2, suggesting that CHHC intervention first modulates lipid-inflammatory pathways and subsequently expands to energy metabolism pathways.

The Sankey diagram illustrated the correspondence between differential metabolites and enriched pathways, with multiple metabolites simultaneously participating in multiple pathways, forming a complex metabolic regulatory network. Phosphatidylcholine metabolites participated in both glycerophospholipid metabolism and sphingolipid metabolism pathways, while arachidonic acid and its derivatives were concentrated in arachidonic acid metabolism and inflammation-related pathways. A metabolomics systematic review has shown that alterations in lipid metabolism and amino acid metabolism are common metabolic features of pregnancy-related diseases, such as fetal growth restriction (30). Postpartum uterine involution involves local inflammation resolution, tissue remodeling, and energy metabolism adjustment, and the metabolic pathways regulated by CHHC intervention are highly consistent with these physiological processes. It has been reported that the metabolome characteristics of women with preterm delivery following cervical cerclage involve significant alterations in lipid metabolism and amino acid metabolism (31), partially overlapping with the metabolic effects of CHHC intervention found in this study, suggesting that TCM external therapies may promote postpartum recovery through similar metabolic pathways.

### 3.6 Correlation analysis between metabolites and clinical indicators and biomarker screening

**Figure 5:**
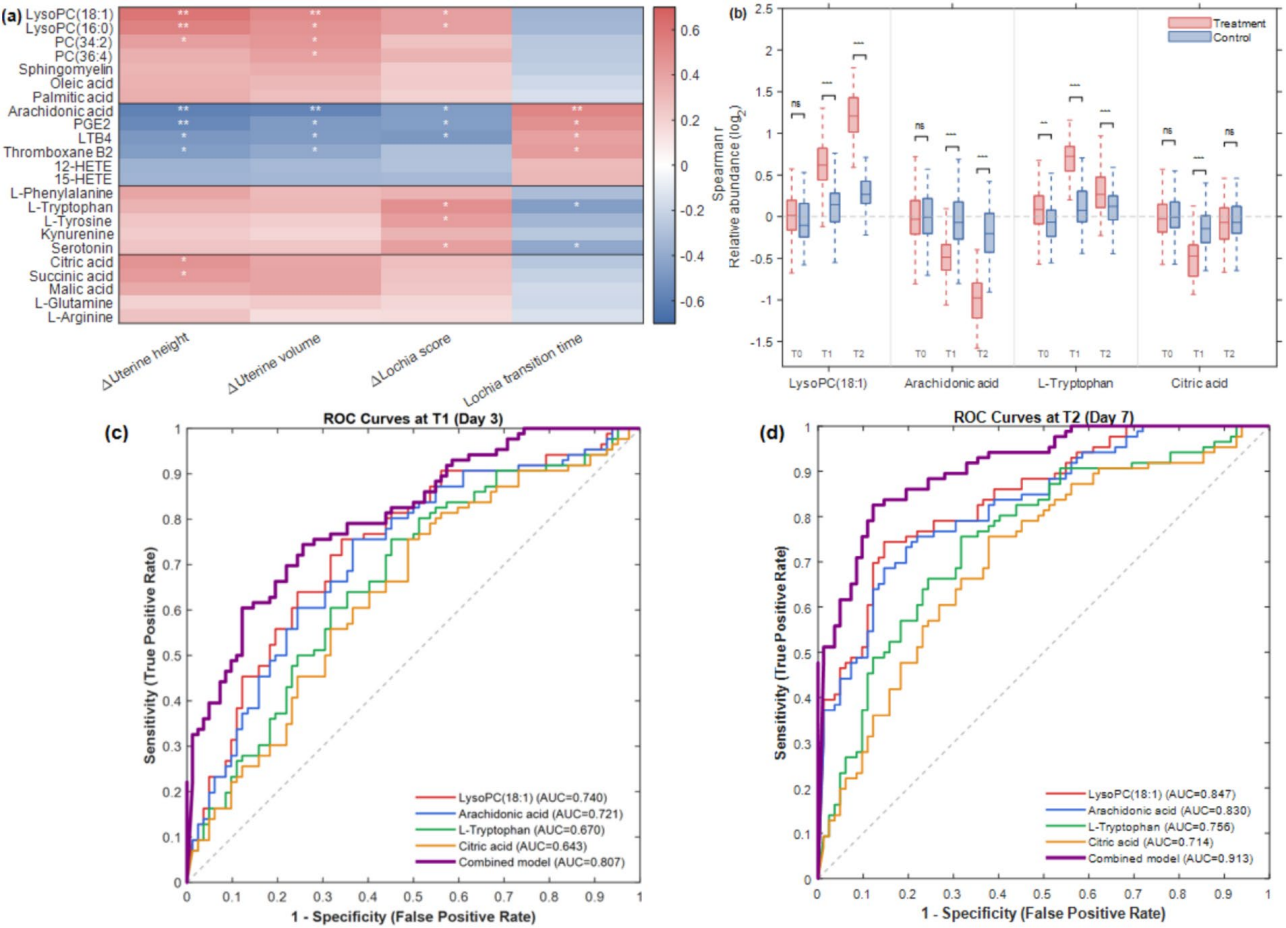
Correlation analysis and candidate biomarker evaluation. **(a)** Spearman correlation heatmap between 21 core differential metabolites and clinical indicators; *p < 0.05, **p < 0.01. **(b)** Box plots showing dynamic changes of four candidate biomarkers at T0, T1, and T2; significance between groups: ns, *p* ≥ 0.05; *p < 0.05; **p < 0.01; ***p < 0.001 (Mann–Whitney *U* test). **(c,d)** ROC curves of candidate markers at T1 **(c)** and T2 **(d)**; individual and four-marker combined model AUCs are shown in each panel. Red: CHHC group (*n* = 86); Blue: routine care group (*n* = 82).

To ensure the reliability of the metabolite–clinical outcome associations, Spearman correlation analysis was performed at both treatment stages: ΔH (T1 − T0) with metabolite changes at T1, and ΔH (T2 − T0) with metabolite changes at T2 (Figure 5a). Comparison of correlation directions between the two stages revealed that 21 of the 23 core metabolites showed consistent correlation directions at both T1 and T2 (Supplementary Table S2). Two metabolites, sphinganine (T1: *r* = 0.085, *p* = 0.31; T2: *r* = −0.142, *p* = 0.08) and L-palmitoylcarnitine (T1: *r* = −0.076, *p* = 0.38; T2: *r* = 0.118, *p* = 0.12), showed opposite but non-significant correlations at both stages and were therefore excluded from subsequent analyses. The following correlation analysis and biomarker screening were based on the remaining 21 core metabolites (Figure 5a). At the T1 stage, ΔH (T1 − T0) showed significant correlations with 15 of the 21 core metabolites (*p* < 0.05). Among them, LysoPC(18:1) (*r* = 0.38, *p* < 0.01) and LysoPC(16:0) (*r* = 0.33, *p* < 0.01) showed positive correlations, while arachidonic acid (*r* = −0.41, *p* < 0.001) and PGE2 (*r* = −0.36, *p* < 0.01) showed negative correlations with uterine fundal height reduction, consistent with the directions observed at T2. The change in uterine fundal height (ΔH, T2-T0) showed significant positive correlations with Cluster 1 metabolites, with LysoPC(18:1) showing the highest correlation coefficient (*r* = 0.58, *p* < 0.001), followed by LysoPC(16:0) (*r* = 0.52, *p* < 0.001), suggesting that phospholipid metabolism improvement is closely related to the progression of uterine involution. Cluster 2 metabolites showed negative correlations with uterine fundal height change, with arachidonic acid (*r* = −0.61, *p* < 0.001) and PGE2 (*r* = −0.55, *p* < 0.001) showing the strongest correlations, indicating that decreased levels of inflammatory mediators are beneficial for the recovery of uterine contraction. Improvement in lochia score (ΔLochia) was significantly correlated with tryptophan metabolism pathway metabolites: L-tryptophan (*r* = 0.47, *p* < 0.01) and serotonin (*r* = 0.44, *p* < 0.01) peaked in the early treatment phase (T1) and then declined, and their expression changes were closely associated with accelerated lochia resolution, suggesting that early activation of tryptophan metabolism may participate in the initiation of postpartum tissue repair. Tricarboxylic acid cycle metabolites citric acid and succinic acid showed positive correlations with uterine volume reduction rate (*r* = 0.39–0.45, *p* < 0.05), reflecting the supportive role of energy metabolism recovery in postpartum uterine involution.

Based on the correlation analysis results, four metabolites with the strongest correlations to clinical indicators were selected as candidate biomarkers: LysoPC(18:1), arachidonic acid, L-tryptophan, and citric acid. Box plots illustrated the dynamic changes of these four metabolites at T0, T1, and T2 (Figure 5b). At baseline (T0), no significant between-group differences were observed for any of the four biomarkers (all *p* > 0.05). As treatment progressed, the between-group differences became progressively significant: at T1, LysoPC(18:1) and arachidonic acid showed significant differences (*p* < 0.01), while L-tryptophan and citric acid reached *p* < 0.05, and at T2, all four biomarkers showed highly significant between-group differences (LysoPC(18:1) and arachidonic acid: *p* < 0.001; L-tryptophan and citric acid: *p* < 0.01). LysoPC(18:1) showed a sustained increase from T0 to T2 in the CHHC group, while no significant change was observed in the routine care group, with the between-group difference reaching the maximum at T2 (*p* < 0.001). Arachidonic acid showed a sustained decreasing trend in the CHHC group, with an approximately 45% decrease at T2 compared to T0, while the routine care group showed only approximately 18% decrease. Studies have demonstrated that metabolomics combined with machine learning can effectively identify high-performance biomarkers for disease diagnosis and prognosis (32).

ROC curve analysis was performed at both T1 and T2 to evaluate the diagnostic performance of candidate markers in distinguishing the CHHC group from the routine care group and to verify the consistency of their discriminatory performance across treatment stages (Figures 5c,d). At T1, the four individual biomarkers showed moderate diagnostic performance: LysoPC(18:1) (AUC = 0.740, 95% CI: 0.652–0.818), arachidonic acid (AUC = 0.721, 95% CI: 0.633–0.803), L-tryptophan (AUC = 0.670, 95% CI: 0.583–0.761), and citric acid (AUC = 0.643, 95% CI: 0.554–0.736). The four-marker combined model yielded an AUC of 0.807 (95% CI: 0.741–0.883) at T1 (Figure 5c). At T2, LysoPC(18:1) achieved an AUC of 0.847 (95% CI: 0.782–0.912), with a sensitivity of 78.5% and specificity of 81.2%; arachidonic acid achieved an AUC of 0.830 (95% CI: 0.763–0.899), with a sensitivity of 76.8% and specificity of 79.5%; L-tryptophan achieved an AUC of 0.756 (95% CI: 0.679–0.833); and citric acid achieved an AUC of 0.714 (95% CI: 0.631–0.793). The combined diagnostic model constructed with all four markers achieved an AUC of 0.913 (95% CI: 0.867–0.959), with a sensitivity of 85.3% and specificity of 87.8%, significantly outperforming individual markers.

Comparison of ROC performance between T1 and T2 revealed that all four biomarkers showed consistently increasing AUC values (Supplementary Table S3), with the combined model AUC improving from 0.807 to 0.913, demonstrating progressively enhanced diagnostic accuracy with treatment duration. This cross-stage consistency in correlation directions (Supplementary Table S2), between-group significance (Figure 5b), and diagnostic performance supports the reliability of the four candidate biomarkers as indicators of CHHC therapeutic response.

## 4 Discussion

### 4.1 Biological significance of key metabolites

This study revealed the dynamic changes in serum metabolic profiles of patients with postpartum uterine subinvolution during CHHC intervention through longitudinal metabolomics analysis. A total of 83 differential metabolites were identified between the CHHC group and the routine care group at T2, among which 21 exhibited continuous changes at both T1 and T2, representing the core metabolic targets of CHHC intervention. Metabolomics, as an important component of systems biology, can reflect the physiological and pathological states of the body at the small-molecule metabolite level, providing new technical approaches for elucidating the mechanisms of action of TCM (33).

Lipid metabolites accounted for the highest proportion among the differential metabolites, with phosphatidylcholines and lysophosphatidylcholines in particular showing sustained upregulation. LysoPC(18:1) exhibited a correlation coefficient of 0.58 with uterine fundal height change, suggesting its potential involvement in regulating uterine smooth muscle contractile function. As an important intermediate product of cell membrane phospholipid metabolism, lysophosphatidylcholine possesses physiological functions, such as regulating vascular tone and promoting smooth muscle contraction. Postpartum uterine involution depends on effective contraction of uterine smooth muscle, and CHHC intervention may enhance uterine contractility by promoting phospholipid metabolism remodeling. Studies have demonstrated that the integrated analysis of metabolomics and lipidomics helps to deeply understand drug targets and metabolic regulatory networks (34).

A key finding of this study is the dynamic nature of CHHC-induced metabolic regulation. The metabolic differences between groups progressively intensified from T0 to T2, as evidenced by increasing OPLS-DA model parameters (*R*^2^*Y*: 0.35 → 0.79 in ESI+), expanding numbers of differential metabolites (47 at T1 vs. 83 at T2), broadening pathway enrichment (8 pathways at T1 vs. 12 at T2), strengthening metabolite–clinical correlations (Supplementary Table S2), and improving biomarker diagnostic performance (combined AUC: 0.807 at T1 vs. 0.913 at T2). This consistent dynamic pattern across all analytical levels is consistent with cumulative pharmacological effects of CHHC therapy, although contributions from non-pharmacological factors cannot be excluded in the absence of a placebo control.

Arachidonic acid and its downstream metabolic products (PGE2, LTB4, thromboxane B2, etc.) showed sustained downregulation in the CHHC group and were significantly negatively correlated with uterine fundal height change. The arachidonic acid metabolic pathway is the core regulatory hub of inflammatory responses, and its metabolic products, such as prostaglandins and leukotrienes, participate in inflammatory mediator release, vascular permeability changes, and tissue damage processes. Postpartum uterine subinvolution is often accompanied by prolonged local inflammatory responses, and the thermal stimulation of CHHC, combined with the active constituents of the herbal medicines, may synergistically inhibit the arachidonic acid cascade, thereby accelerating inflammation resolution and tissue repair. It has been reported that menstrual blood lipidomics analysis can effectively identify diagnostic markers for endometriosis, and alterations in arachidonic acid metabolism represent a common metabolic feature of gynecological inflammatory diseases (35).

Tryptophan metabolism pathway metabolites (L-tryptophan, serotonin) were significantly correlated with lochia score improvement. The tryptophan–serotonin metabolic axis is involved not only in neurotransmitter synthesis but also possesses functions in regulating immune function and tissue repair. Normal resolution of postpartum lochia reflects the repair process of the endometrium, and improvement in tryptophan metabolism may accelerate lochia resolution by promoting the reestablishment of local immune homeostasis. Changes in tricarboxylic acid cycle metabolites (citric acid, succinic acid, and malic acid) reflect adjustments in energy metabolism status. As a high-energy-consuming physiological process, uterine involution requires an adequate energy supply to support cell proliferation and tissue remodeling.

### 4.2 Metabolic pathways and the “warming meridians and activating blood” mechanism

KEGG pathway enrichment analysis revealed that arachidonic acid metabolism, glycerophospholipid metabolism, sphingolipid metabolism, amino acid metabolism, and the tricarboxylic acid cycle were significantly enriched, and alterations in these pathways correspond organically to the traditional efficacy of CHHC in “warming meridians and activating blood.” According to TCM theory, postpartum uterine subinvolution is mostly caused by cold coagulation and blood stasis with impaired qi and blood circulation. CHHC exerts the effects of warming meridians to dispel cold and activating blood to resolve stasis through thermal stimulation and transdermal absorption of herbal medicines. The metabolomics results provide a modern biological interpretation for this traditional theory at the molecular level.

Regarding the route of action, CHHC involves direct application of the herbal compress to the skin surface of the lower abdomen and lumbosacral region at 40–50 °C for 20–30 min. The active constituents of the herbal medicines may enter the body primarily through transdermal absorption. Heat application increases local skin temperature, dilates dermal capillaries, enhances skin permeability, and facilitates the penetration of lipophilic active compounds across the stratum corneum into the systemic circulation (36). In addition, several herbs in the formula, particularly *Artemisia argyi* and *Ligusticum chuanxiong*, contain volatile aromatic compounds (e.g., eucalyptol and ligustilide) that are released upon heating. Inhalation of these volatile compounds may constitute a secondary route of systemic exposure. Studies have demonstrated that inhaled plant-derived volatile compounds can be absorbed through the respiratory mucosa and exert measurable effects on autonomic nervous function and metabolic parameters (37). Therefore, the metabolic changes observed in this study may result from the combined effects of transdermal absorption, local thermal stimulation, and potential inhalation of volatile aromatic compounds. However, the relative contribution of each route cannot be determined from the current study design and warrants further investigation.

The “warming meridians” effect may be closely related to the activation of energy metabolism pathways. The tricarboxylic acid cycle is the core hub of cellular aerobic metabolism, and dynamic changes in intermediate metabolites, such as citric acid and succinic acid, reflect mitochondrial functional status. The thermal stimulation of CHHC can increase local tissue temperature, enhance enzyme activity and metabolic rate, thereby promoting energy production. The energy metabolism changes observed in this study may represent the adaptive response of uterine tissue to the thermal component of CHHC intervention.

The “activating blood” effect is related to the regulation of lipid metabolism and inflammatory pathways. Alterations in glycerophospholipid metabolism and sphingolipid metabolism pathways affect cell membrane fluidity and signal transduction function, and upregulation of phosphatidylcholine metabolites is beneficial for maintaining cell membrane structural integrity and promoting cell proliferation. Inhibition of the arachidonic acid metabolic pathway reduces the generation of pro-inflammatory mediators and alleviates local inflammatory responses. The pathological state of “blood stasis” in TCM overlaps with modern medical concepts, such as microcirculation disorders and inflammatory responses. The herbal medicines in the CHHC formula, such as *Artemisia argyi, Carthamus tinctorius, Angelica sinensis, Ligusticum chuanxiong*, and *Leonurus japonicus*, all possess the efficacy of activating blood and resolving stasis, and their pharmacologically active constituents—such as ferulic acid from *Angelica sinensis*, hydroxysafflor yellow A from *Carthamus tinctorius*, and stachydrine from *Leonurus japonicus*—may exert synergistic effects by targeting lipid metabolism and amino acid metabolism (28), suggesting that different TCM external therapies may exert therapeutic effects through common metabolic pathways.

**Figure 6:**
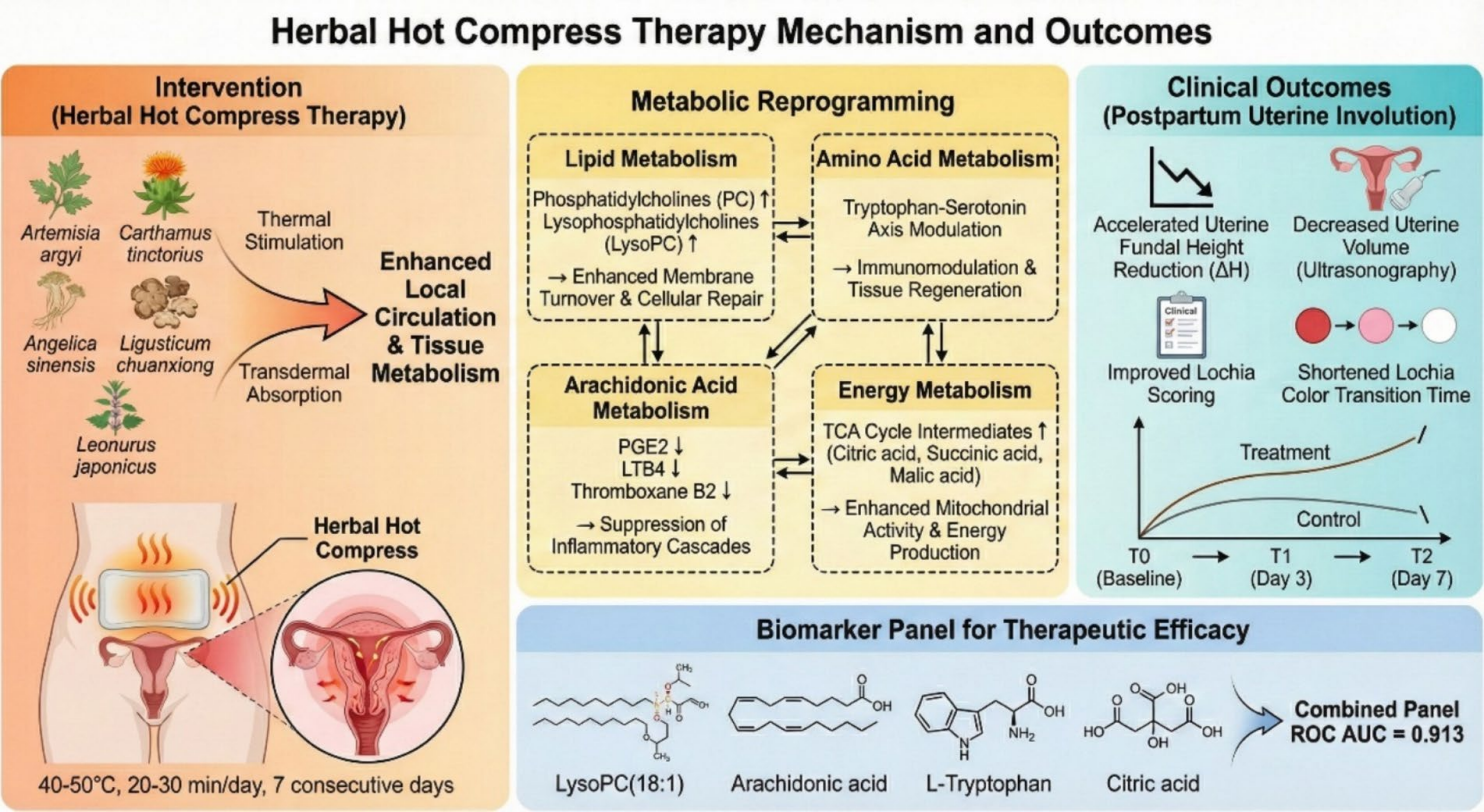
Schematic diagram of metabolic regulatory mechanisms underlying herbal hot compress-promoted postpartum uterine involution.

The four typical dynamic patterns of metabolite changes observed in this study provide temporal-dimension evidence for understanding the mechanisms of action of CHHC. Metabolites with sustained patterns (such as LysoPC and arachidonic acid classes) suggest cumulative pharmacological effects that intensify with treatment duration, whereas rise-then-fall or fall-then-rise patterns (such as tryptophan metabolites and TCA cycle intermediates) may reflect stage-specific regulatory processes, with early activation initiating tissue repair followed by homeostatic readjustment. These distinct temporal trajectories indicate that CHHC intervention does not uniformly alter all metabolic pathways but rather engages different metabolic networks in a temporally coordinated manner. Longitudinal metabolomics studies have demonstrated that time-series sampling designs can capture the dynamic metabolic effects of interventions (27), and the three-time-point design of this study provides important evidence for revealing the temporal characteristics of metabolic regulation by CHHC intervention. The integrated mechanistic framework linking the intervention, the key metabolic pathways, and the clinical outcomes discussed above is summarized schematically in Figure 6.

### 4.3 Clinical translation value

The four candidate biomarkers identified in this study (LysoPC(18:1), arachidonic acid, L-tryptophan, and citric acid) demonstrated good diagnostic performance, with the four-marker combined model achieving an AUC of 0.913, sensitivity of 85.3%, and specificity of 87.8%. These metabolic markers are expected to provide metabolic-level evidence for the objective evaluation of CHHC intervention efficacy, compensating for the current limitations of relying primarily on subjective indicators such as ultrasonographic examination and symptom scoring.

Importantly, the four candidate biomarkers demonstrated consistent trends with clinical efficacy at both T1 and T2 stages: their between-group differences increased progressively, their correlations with uterine involution remained directionally consistent (Supplementary Table S2), and their ROC diagnostic performance improved from T1 to T2 (Supplementary Table S3). This cross-stage consistency strengthens the reliability of these biomarkers as indicators of CHHC therapeutic response (Table 3).

**Table 3:** Clinical efficacy evaluation at T2.

Efficacy	CHHC group (*n* = 86)	Routine care group (*n* = 82)
Markedly effective	48 (55.8%)	25 (30.5%)
Effective	30 (34.9%)	34 (41.5%)
Ineffective	8 (9.3%)	23 (28.0%)
Total effective rate	90.7%	72.0%

Total effective rate = (markedly effective + effective)/total × 100%. Between-group comparison: *χ*^2^ = 9.56, *p* < 0.01.

Metabolomics has made important progress in the field of biomarker discovery for gynecological diseases (38, 39). Untargeted metabolomics approaches have also been applied to identify diagnostic biomarkers in other clinical conditions, such as psoriasis vulgaris (40) and generalized ligamentous laxity (41), further demonstrating the broad applicability of this technology. Studies have shown that the identification of ovarian function-related metabolic markers provides new tools for infertility diagnosis and treatment monitoring (38). Metabolomics studies on poor ovarian response have also identified metabolic markers with diagnostic and prognostic value (39). This study applied metabolomics to the efficacy evaluation of TCM external therapies, expanding the application scope of metabolomics in TCM research.

From a clinical translation perspective, the findings of this study have the following potential applications: the identified biomarkers can provide molecular indicators for the objective evaluation of CHHC efficacy, the revealed metabolic pathways provide scientific evidence for understanding the mechanisms of action of CHHC, and the established multi-marker combined diagnostic model provides methodological reference for efficacy prediction of TCM external therapies. These findings contribute to promoting the standardization and modernization of TCM and provide a reference for evidence-based medicine research on TCM external therapies.

### 4.4 Study limitations and future directions

This study has the following limitations. This study was an observational cohort study rather than a randomized controlled trial, with grouping based on actual clinical treatment, potentially introducing selection bias; no placebo control (e.g., hot compress with inert materials) was established, making it difficult to completely separate the pharmacological effects of the herbal ingredients from the thermal and pressure stimulation of the compress itself. From a practical standpoint, effective blinding was infeasible due to the distinct herbal odor of CHHC, and from an ethical perspective, applying a known ineffective compress to patients with confirmed uterine subinvolution raised concerns during ethics review. However, several lines of evidence suggest that the observed metabolic changes are not solely attributable to physical stimulation: first, the metabolic profiles of the two groups were highly comparable at baseline (*R*^2^*Y* = 0.35, *Q*^2^ = 0.12), indicating that the subsequent metabolic divergence was not driven by pre-existing group differences; second, the identified differential metabolites were predominantly enriched in arachidonic acid metabolism and glycerophospholipid metabolism pathways, which are known pharmacological targets of active constituents in the CHHC formula, such as ferulic acid from *Angelica sinensis* and hydroxysafflor yellow A from *Carthamus tinctorius* (28); third, the pathway-specific and progressive metabolic divergence from T0 to T2 is more consistent with cumulative pharmacological action than with nonspecific responses to thermal or mechanical stimulation, which would be expected to produce acute, transient effects; and blinding was not employed for clinical outcome assessment, potentially introducing measurement bias. Additionally, the single-center study design limits the generalizability of conclusions; the sample size was relatively limited, and an independent validation cohort was lacking. Metabolite identification was not validated with authentic standards, and some structural annotations require further confirmation; furthermore, the candidate biomarkers were identified through untargeted metabolomics, which provides comprehensive profiling but limited quantitative precision, and their diagnostic thresholds were not independently validated using targeted quantitative methods; mechanistic discussions were primarily based on pathway enrichment analysis and literature inference, lacking direct validation from cell and animal experiments.

Given these limitations, the present findings should be interpreted as hypothesis-generating rather than confirmatory, and the metabolic associations reported herein require validation in future placebo-controlled trials. Future research directions include the following: designing randomized controlled trials with a three-arm protocol (CHHC group, thermal compress alone group, and routine care group) to isolate the specific pharmacological contribution of herbal ingredients; conducting multi-center, large-sample prospective validation studies to confirm the diagnostic performance and clinical applicability of candidate biomarkers; employing targeted quantitative methods, such as multiple reaction monitoring (MRM)-based LC–MS/MS, enzyme-linked immunosorbent assay (ELISA), or colorimetric assays for precise quantification and independent validation of the candidate biomarkers in external clinical cohorts; integrating multi-omics data, such as transcriptomics and proteomics to construct more complete CHHC mechanism of action networks; conducting *in vitro* cell experiments and animal model studies to verify causal relationships of key metabolic pathways; and exploring the dose-effect relationships between individual herbal components in the CHHC formula and metabolic regulatory effects to provide evidence for formula optimization.

## 5 Conclusion

This study employed a longitudinal metabolomics approach based on UPLC-Q-TOF-MS to systematically reveal the dynamic changes in serum metabolic profiles of patients with postpartum uterine subinvolution during CHHC intervention. The study found that the metabolic profiles of the CHHC group and the routine care group were highly overlapping at baseline, gradually diverged as treatment progressed, and showed the most pronounced separation at day 7 of treatment. A total of 83 differential metabolites were identified at T2, among which 21 exhibited continuous changes at both T1 and T2, presenting four typical dynamic patterns: sustained increase, sustained decrease, rise-then-fall, and fall-then-rise. KEGG pathway enrichment analysis revealed that the differential metabolites were primarily enriched in arachidonic acid metabolism, glycerophospholipid metabolism, sphingolipid metabolism, amino acid metabolism, and the tricarboxylic acid cycle. Alterations in these metabolic pathways correspond organically to the traditional efficacy of CHHC in “warming meridians and activating blood,” providing a preliminary modern biological interpretation for TCM theory at the molecular level.

Based on correlation analysis and ROC curve evaluation, this study identified four candidate biomarkers—LysoPC(18:1), arachidonic acid, L-tryptophan, and citric acid—with the four-marker combined model achieving an AUC of 0.913, demonstrating good diagnostic performance. These biomarkers are expected to provide metabolic-level molecular indicators for the objective evaluation of CHHC efficacy. This study has limitations, such as a single-center design, a limited sample size, and the lack of an independent validation cohort. Future studies should conduct multi-center validation research and mechanistic verification experiments. In summary, this study is the first to characterize the metabolic regulatory characteristics of CHHC in the context of postpartum uterine involution from a dynamic metabolomics perspective, providing a new research paradigm for the scientific interpretation and evidence-based evaluation of TCM external therapies.

## Data Availability

The raw data supporting the conclusions of this article will be made available by the authors, without undue reservation.
