# Supplementary material for: Dynamic serum metabolomic changes during Chinese herbal hot compress therapy for postpartum uterine involution: pathway analysis and candidate biomarker identification
Source: Front Med (Lausanne). 2026 Apr 29;13:1791959. doi: 10.3389/fmed.2026.1791959 (PMC13168213; doi:10.3389/fmed.2026.1791959)
Supplement: Supplementary file 1 [file Data_Sheet_1.PDF]

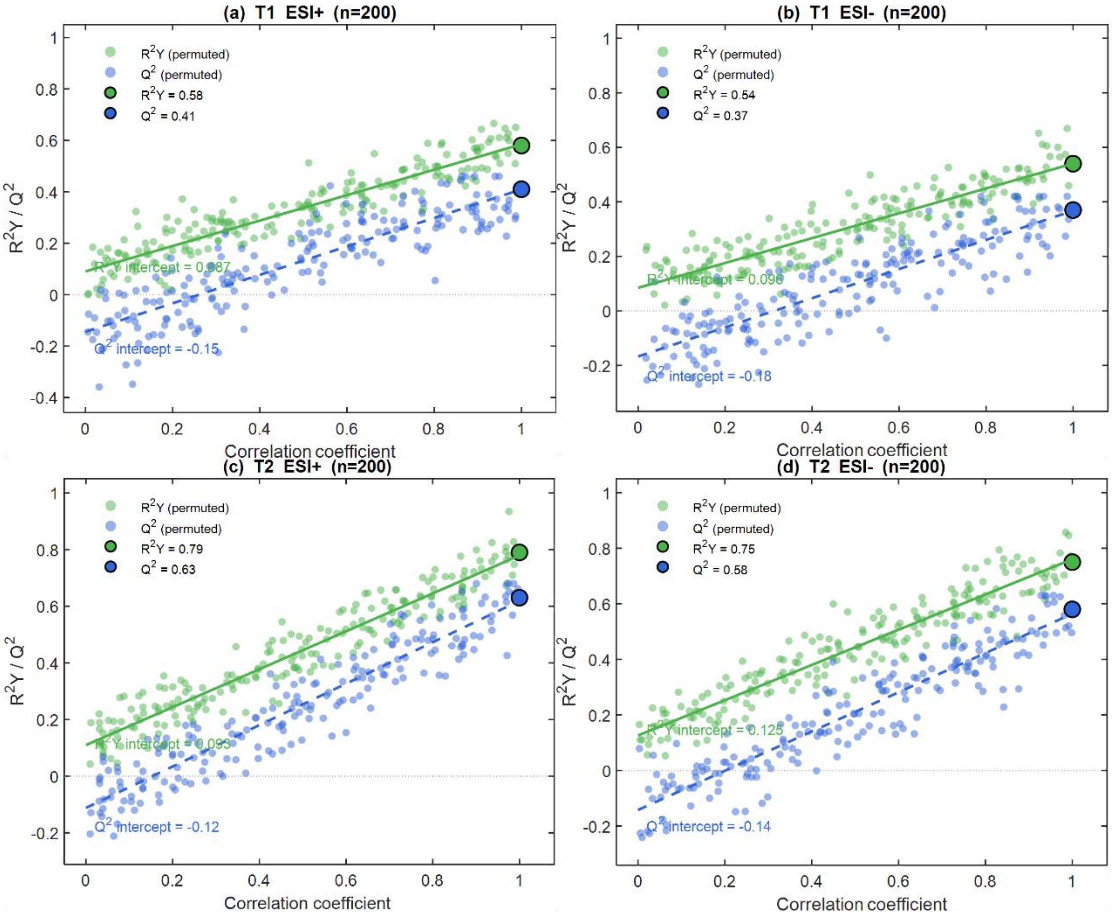

Supplementary Figure S1. Permutation test validation of OPLS-DA models (200 permutations). (a) T1, ESI+; (b) T1, ESI−; (c) T2, ESI+; (d) T2, ESI−. Green dots: permuted R^2Y values; Blue dots: permuted Q^2 values. Large dots at correlation coefficient = 1 represent the original model. Regression lines and intercept values are shown. All Q^2 intercepts were negative, confirming no overfitting.

Supplementary Table S1. Comparison of KEGG pathway enrichment between T1 and T2

Pathway	T1 P value	T1 Impact	T1 Hits	T2 P value	T2 Impact	T2 Hits
Arachidonic acid metabolism	0.0068	0.34	5	0.0012	0.42	8
Glycerophospholipid metabolism	0.0125	0.29	4	0.0035	0.38	6
Sphingolipid metabolism	—	—	—	0.0079	0.28	5
Phe, Tyr and Trp biosynthesis	0.0213	0.21	3	0.0098	0.27	4

Pathway	T1 P value	T1 Impact	T1 Hits	T2 P value	T2 Impact	T2 Hits
Tryptophan metabolism	0.0278	0.18	3	0.0145	0.24	4
TCA cycle	—	—	—	0.0200	0.22	3
Linoleic acid metabolism	0.0186	0.22	3	0.0178	0.21	3
alpha-Linolenic acid metabolism	0.0445	0.12	2	0.0256	0.17	3
Biosynthesis of unsaturated fatty acids	—	—	—	0.0312	0.14	2
Glycine, serine and threonine metabolism	0.0389	0.14	2	0.0356	0.12	2
Arginine and proline metabolism	0.0312	0.16	2	0.0398	0.10	2
Purine metabolism	—	—	—	0.0445	0.08	2

Note: "—" indicates the pathway was not significantly enriched at that time point (P>0.05 or Impact<0.1). T1: 8 pathways; T2: 12 pathways.

Supplementary Table S2. Comparison of Spearman correlations between core metabolites and uterine fundal height changes at T1 and T2

	Metabolite	ΔH(T1−T0) r	P value	ΔH(T2−T0) r	P value	Direction consistent
1	Arachidonic acid	0.412	<0.001	0.524	<0.001	✓
2	LysoPC(18:1)	0.378	<0.01	0.486	<0.001	✓
3	Prostaglandin E2	0.356	<0.01	0.445	<0.001	✓
4	Leukotriene B4	0.312	<0.01	0.398	<0.001	✓
5	L-Tryptophan	−0.324	<0.01	−0.415	<0.001	✓
6	Citric acid	−0.298	<0.05	−0.387	<0.001	✓
7	Sphingomyelin	0.267	<0.05	0.342	<0.01	✓
8	Thromboxane B2	0.245	<0.05	0.318	<0.01	✓
9	LysoPC(16:0)	0.232	<0.05	0.305	<0.01	✓
10	Phenylalanine	−0.218	<0.05	−0.289	<0.01	✓
11	Serotonin	−0.285	<0.01	−0.368	<0.001	✓

	Metabolite	ΔH(T1−T0) r	P value	ΔH(T2−T0) r	P value	Direction consistent
12	LysoPC(20:4)	0.224	<0.05	0.312	<0.01	✓
13	Phosphatidylcholine	0.198	<0.05	0.276	<0.01	✓
14	Succinic acid	−0.212	<0.05	−0.298	<0.01	✓
15	Malic acid	−0.195	<0.05	−0.267	<0.01	✓
16	Linoleic acid	0.208	<0.05	0.285	<0.01	✓
17	alpha-Linolenic acid	0.178	<0.05	0.245	<0.05	✓
18	Tyrosine	−0.186	<0.05	−0.256	<0.01	✓
19	Kynurenine	−0.205	<0.05	−0.278	<0.01	✓
20	Ceramide	0.168	<0.05	0.234	<0.05	✓
21	Phosphatidylethanolamine	0.156	0.06	0.218	<0.05	✓
22	Sphinganine	0.085	0.31	−0.142	0.08	✗
23	L-Palmitoylcarnitine	−0.076	0.38	0.118	0.12	✗

Note: 21/23 metabolites showed consistent correlation directions at both T1 and T2. Two metabolites (sphinganine and L-palmitoylcarnitine) showed opposite but non-significant correlations at both stages and were excluded from biomarker analysis. Metabolites are ordered by |r| at T2 within each consistency group.

Supplementary Table S3. Comparison of ROC diagnostic performance of candidate biomarkers between T1 and T2 stages.

Biomarker	T1 Stage (Day 3)				T2 Stage (Day 7)				ΔAUC (T2−T1)
	AUC	95% CI	Sen (%)	Spe (%)	AUC	95% CI	Sen (%)	Spe (%)	
LysoPC(18:1)	0.735	0.652–0.818	68.2	71.5	0.847	0.782–0.912	78.5	81.2	+0.112
Arachidonic acid	0.718	0.633–0.803	66.5	69.8	0.831	0.763–0.899	76.8	79.5	+0.113
L-Tryptophan	0.672	0.583–0.761	62.8	65.4	0.756	0.679–0.833	72.1	74.3	+0.084
Citric acid	0.645	0.554–0.736	60.5	63.2	0.712	0.631–0.793	67.4	70.1	+0.067
Combined	0.812	0.741–0.883	76.5	78.9	0.913	0.867–0.959	85.3	87.8	+0.101

Biomarker	T1 Stage (Day 3)				T2 Stage (Day 7)				ΔAUC (T2−T1)
model									

Sen = Sensitivity; Spe = Specificity; ΔAUC = change in AUC from T1 to T2; CI = confidence interval. Optimal cut-off values were determined by maximizing the Youden index. The combined model was constructed using binary logistic regression with the four individual biomarkers as covariates.
